# Prof. Ashurst on Anæsthetics

**Published:** 1879-02

**Authors:** 


### Miscellaneous.

### PROF. ASHURST ON ANÆSTHETICS.

Upon the subject of anæsthetics the author makes some judicious suggestions. He discourages the reckless use of them, he would not employ them except for important operations.

Upon the question of safety, the comparative safety of chloroform and ether, he says: "I prefer ether in a very large majority of cases; it is certainly safer than chloroform, and is sufficiently convenient for almost every case that a surgeon is called upon to treat."

It is seldom that patients die through the head under anæsthesia. Our author says: "Death from the administration of an anæsthetic may come from the failure of either foot of the vital tripod, the head, the lungs, or the heart; in other words, it may be due to *coma* to *asphyxia* or to *syncope*." Recent experiments show conclusively that in animals destroyed by chloroform the heart fails first, in those in whom ether is administered to the fatal point, that death occurs through the lungs. This corresponds with clinical experience, it is rare to see a patient die from coma.

This furnishes also the practical solution of the question of safety—syncope is a much more dangerous condition than asphyxia. When the heart is smitten by chloroform it is usually fatal; when, however, the lungs are embarrassed by ether, the withdrawal of the agent for a moment is sufficient to banish danger; a few inspirations of air, places the person in a condition of safety.

In both of the patients which it was the misfortune of the writer to lose, under chloroform, the heart failed, promptly and fatally in the first, in the second it failed, rallied, but in a moment ceased to pulsate. The field of the operation in each became suddenly bloodless, showing the condition of the heart. Under ether when respiration becomes labored, jerky, or sonorous, the blood still follows the knife, respiration is oppressed, but the circulation is still intact.

Death does not occur from the quantity of or from the manner in which an anæsthetic is administered, but from the peculiar condition of the patient at that hour. In my first death the chloroform was dropped upon a flannel ladle, held one or two inches from the face, allowing the freest circulation of air. Not two drachms were used. A lady, who died a few years ago in a dentist's chair, after taking but a few inhalations of chloroform, passing from life, not from sleep, to death, had taken it as she said "a hundred times." The physician, Dr. Stewart, of Bloomingsburg, Fayette Co., Ohio, informed me that he had, on one occasion, kept her continuously under its influence for *twelve hours*.

It is this influence upon the heart that renders chloroform dangerous in Dental practice. Syncope too, is more likely to be final when occurring in the erect than in the horizontal position.

It requires so much time and so much ether to produce anæsthesia in the ordinary manner of administration, that many surgeons have adhered to chloroform on the mere question of convenience, of promptness in the production of the desired result. To avoid this delay, to get a quick condition of insensibility, the patient must be overwhelmed with the ether, all air must be excluded. At the clinics of the writer since the spring of 1874 not, on average, more than five minutes have been consumed in inducing a profound influence. At first Lent's apparatus (a very good one by the way) was used, now a tin funnel bordered with india rubber, to fit the face, and receptacle for a sponge in the upper portion has been substituted. When it does not fit the face closely, the base of it is surrounded by a towel so that the patient shall breathe ether alone.

